# Plant litter dynamics in the forest-stream interface: precipitation is a major control across tropical biomes

**DOI:** 10.1038/s41598-017-10576-8

**Published:** 2017-09-07

**Authors:** Alan M. Tonin, José F. Gonçalves, Paulino Bambi, Sheyla R. M. Couceiro, Lorrane A. M. Feitoza, Lucas E. Fontana, Neusa Hamada, Luiz U. Hepp, Vânia G. Lezan-Kowalczuk, Gustavo F. M. Leite, Aurea L. Lemes-Silva, Leonardo K. Lisboa, Rafael C. Loureiro, Renato T. Martins, Adriana O. Medeiros, Paula B. Morais, Yara Moretto, Patrícia C. A. Oliveria, Evelyn B. Pereira, Lidiane P. Ferreira, Javier Pérez, Mauricio M. Petrucio, Deusiano F. Reis, Renan S. Rezende, Nadia Roque, Luiz E. P. Santos, Ana E. Siegloch, Gabriela Tonello, Luz Boyero

**Affiliations:** 1 Limnology Lab, Dept. of Ecology, IB, Univ. of Brasília (UnB), 70910-900 Brasília, DF Brazil; 2 Faculty of Science and Technology, Univ. of the Basque Country (UPV/EHU), Leioa, Spain; 3 Lab de Ecologia e Taxonomia de Invertebrados, Univ. Federal do Oeste do Pará (UFOPA), Inst. de Ciências e Tecnologia das Águas, Campus Amazônia Boulevard, 68040-470 Santarém, PA Brazil; 4 Univ. Federal de Roraima, Centro de Estudos da Biodiversidade, 69304-000 Boa Vista, RR Brazil; 5 Univ. Regional Integrada do Alto Uruguai e das Missões, Dept. Ciências Biológicas, 99709-910 Erechim, RS Brazil; 6 Coordenação de Biodiversidade, Instituto Nacional de Pesquisas da Amazônia (INPA), 69067-375 Manaus, AM Brazil; 7 Lab. de Ecologia de Águas Continentais, Dept. de Ecologia e Zoologia, Centro de Ciências Biológicas, Univ. Federal de Santa Catarina, 88040-900 Florianópolis, SC Brazil; 8 Univ. Federal da Bahia, Dept. de Botânica, Inst. de Biologia, 40170-115 Salvador, BA Brazil; 9 Lab. Microbiologia Ambiental e Biotecnologia, Univ. Federal do Tocantins, 77001-923 Palmas, TO Brazil; 10 Univ. Federal do Paraná, Dept. de Biodiversidade, 85950-000 Palotina, PR Brazil; 11 IKERBASQUE, Basque Foundation for Science, Bilbao, Spain; 12 College of Marine and Environmental Sciences and TropWater, James Cook Univ, Townsville, QLD 4811 Australia

## Abstract

Riparian plant litter is a major energy source for forested streams across the world and its decomposition has repercussions on nutrient cycling, food webs and ecosystem functioning. However, we know little about plant litter dynamics in tropical streams, even though the tropics occupy 40% of the Earth’s land surface. Here we investigated spatial and temporal (along a year cycle) patterns of litter inputs and storage in multiple streams of three tropical biomes in Brazil (Atlantic forest, Amazon forest and Cerrado savanna), predicting major differences among biomes in relation to temperature and precipitation regimes. Precipitation explained most of litter inputs and storage, which were generally higher in more humid biomes (litterfall: 384, 422 and 308 g m^−2^ y^−1^, storage: 55, 113 and 38 g m^−2^, on average in Atlantic forest, Amazon and Cerrado, respectively). Temporal dynamics varied across biomes in relation to precipitation and temperature, with uniform litter inputs but seasonal storage in Atlantic forest streams, seasonal inputs in Amazon and Cerrado streams, and aseasonal storage in Amazon streams. Our findings suggest that litter dynamics vary greatly within the tropics, but point to the major role of precipitation, which contrasts with the main influence of temperature in temperate areas.

## Introduction

Freshwater ecosystems are widely spread across terrestrial landscapes and receive large amounts of litter from riparian vegetation^1^. In particular, rivers and streams receive, transport and store approximately 2.1 Pg of terrestrial organic carbon each year, which represents a considerable fraction of the overall net ecosystem production of terrestrial ecosystems^2^. Despite their small spatial extent, ﻿permanent﻿﻿﻿ headwater streams significantly contribute to organic matter processing due to their high retentive capacity, constant water flow and high nutrient availability^3, 4^. Organic material – mostly leaf litter – enters streams through two routes^5^, directly by vertical litterfall (hereafter litterfall), or laterally from the forest soil (hereafter lateral inputs), and can be transported downstream by water flow or retained in depositional habitats or structures such as boulders or logs. The retained litter represents an important energy source for stream food webs^6, 7^, and its subsequent decomposition contributes significantly to the global carbon cycle^8^. Thus, quantifying the magnitude and timing of litter inputs and storage in headwater streams seems a major step towards understanding the functioning of ecosystems and the cycling of organic matter globally.

Organic matter inputs and storage in temperate and boreal forest streams have been studied for decades, especially in Europe and North America^1, 9–11^, where the timing and the magnitude of these processes are well known. In contrast, comparable studies in tropical streams are scarce, so most basic questions about natural variation of litter inputs and storage within the tropics remain unknown. For example, are there similarities in the timing of litter inputs to the stream within and across tropical biomes? In which periods of the year does most litter enter and accumulate in streams? The few existing assessments of organic matter inputs and storage in tropical streams have mostly been restricted to single streams^12–14^ or a single region^15, 16^, which limits the identification of spatial and temporal patterns of variation and their main controls at larger scales^17^. Also, ignoring the natural variation of litter inputs and storage in the tropics may limit the understanding of key ecosystem processes such as litter decomposition and secondary production (which in turn depend of litter sources)^7^, challenging the development of an integrated view of tropical stream ecosystems.

Litterfall has been widely used by terrestrial ecologists as a good estimator of plant productivity (i.e., annual net primary productivity), and it is generally positively influenced by temperature, precipitation and soil fertility^18–20^. However, in tropical forests, litterfall annual variability seems to depend mainly on precipitation and solar radiation, with litterfall peaks corresponding to the dry season, which contrasts with most temperate forests, where litter peaks occur in autumn and are predicted by temperature and solar radiation^19^. Lateral litter inputs tend to be less predictable than litterfall, as they depend on multiple factors such as litter accumulation in forest soils, the slope of stream banks, litter humidity – as dry litter is more vulnerable to be transported by the wind^21^ – and physical processes such as overland flow and wind that may enhance litter transport into the stream^22, 23^. Litter storage in the stream depends on both litterfall and lateral inputs, and is mainly determined by water flow conditions (i.e., low-flow streams have lower shear stress)^24, 25^, the stream retention capacity (shallow streams have more retentive structures), which together determine the downstream transport^26^ and the rate at which litter is decomposed which acts as a longer-term control^27^.

**Figure 1:**
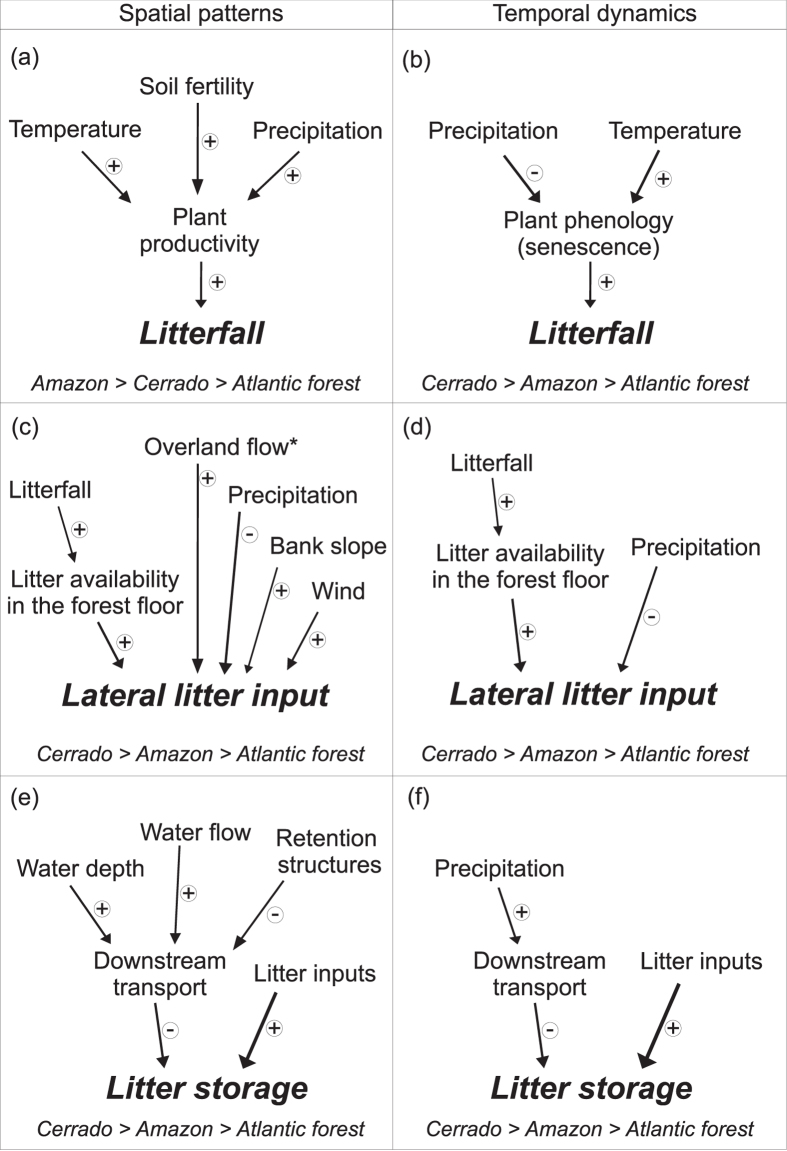
Expected predictors of **(a, c, e)** spatial patterns and **(b, d, f)** temporal dynamics of **(a, b)** litterfall, **(c, d)** lateral inputs and **(e, f)** benthic storage. Plus and minus signs near arrows indicate the direction of effects (positive or negative, respectively). The expectation for the spatial patterns and temporal dynamics of each process is indicated below each process.

**Figure 2:**
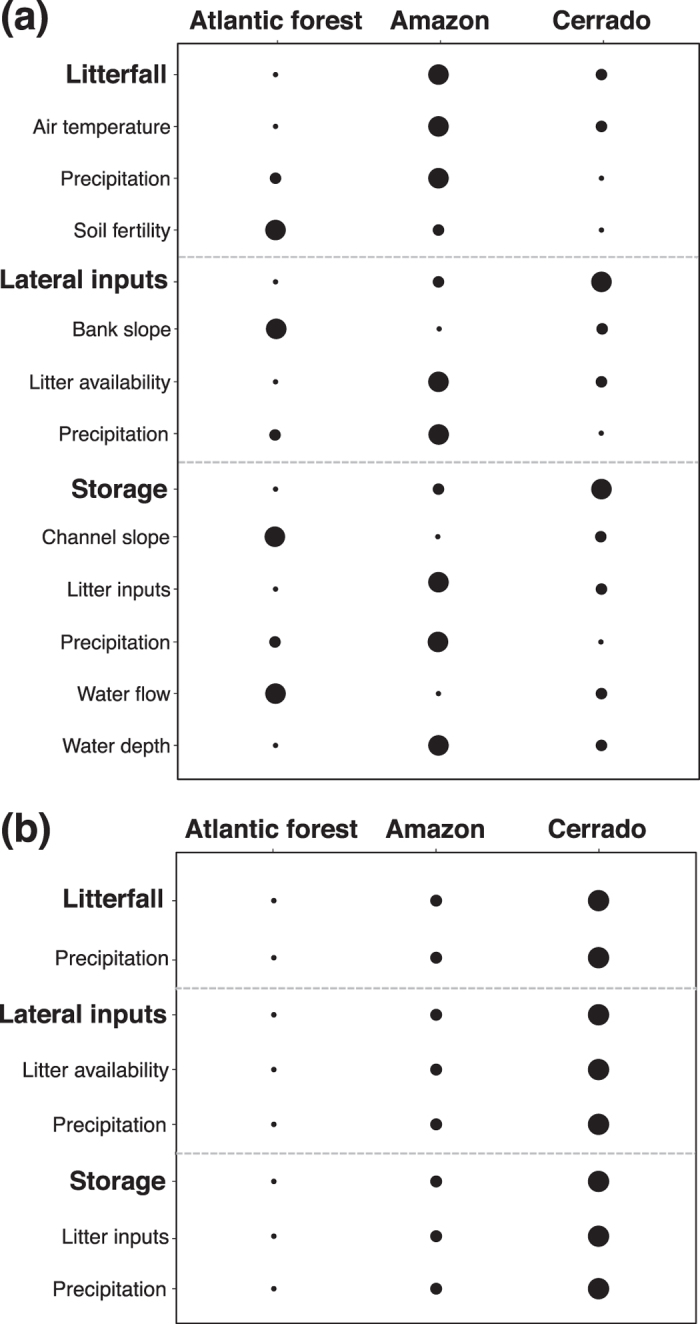
Expected predictors of litterfall, lateral inputs and storage in Atlantic forest, Amazon forest and Cerrado savanna biomes. Circles of different size indicate effects of different magnitude (small, medium and large) for the **(a)** spatial patterns and **(b)** temporal dynamics of each process.

The complexity of biological and environmental interactions involved in litter dynamics and the lack of basic information have precluded robust tests of which factors control litter inputs and storage in tropical streams. Here we addressed this issue in a multi-site field study across three biomes in Brazil (Atlantic forest, Amazon forest and Cerrado savanna) encompassing 30° of latitude (28°S-2°N). We aimed to explore the patterns of litter inputs (divided into two routes: litterfall and lateral inputs) and storage in streams across multiple spatial scales (from within stream to among biomes), as well as temporal dynamics within an annual cycle, and to identify which environmental and biological factors are the main influences on these processes. For that purpose we tested the following hypotheses (Fig. 1): (i) spatial patterns of litterfall would mainly depend on plant productivity (which in turn depends on climatic and soil factors), while its temporal dynamics would mainly depend on plant phenology (in turn related to climate) (Fig. 2); (ii) spatial patterns and temporal dynamics in lateral litter inputs would result from the combined effect of multiple environmental factors (including climatic and other factors) and of litterfall (Fig. 2); (iii) litter storage would vary spatially depending on litter inputs and stream channel characteristics (e.g., retention structures) while temporal dynamics would be greatly influenced by precipitation (Fig. 2); and (iv) the greatest spatial variance of all these processes would occur among biomes, in relation to climatic and geologic variation, with less variance at smaller scales.

## Results

### Litterfall

**Figure 3:**
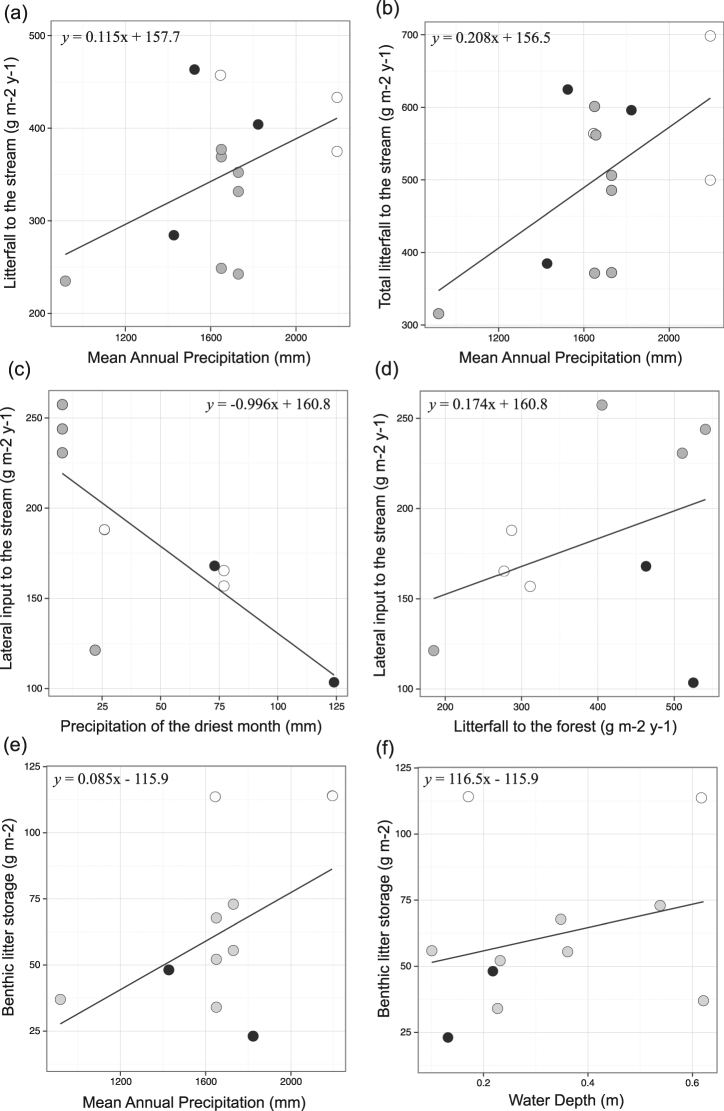
Relationships between litter inputs, benthic storage and their predictors in Atlantic forest (black circles), Amazon (white circles) and Cerrado streams (grey circles): (**a**) litterfall vs. mean annual precipitation (MAP); (**b**) total litterfall vs. MAP; (**c**) lateral inputs vs. precipitation of the driest month (PDM); (**d**) lateral inputs vs. litterfall to the forest; (**e**) storage vs. MAP; and (**f**) storage vs. water depth. Litter inputs are in g per m^2^ per year and storage in g per m^2^.

Litterfall was 20% higher in Atlantic forest and 40% higher in Amazon than in Cerrado, but similar between Atlantic forest and Amazon (mean ± SE in Amazon, Atlantic forest and Cerrado, respectively: 384 ± 43, 422 ± 20 and 308 ± 22 g leaf dry mass m^−2^ year^−1^; Table S3; Fig. S3). Litterfall accounted for 72 ± 13% in Atlantic forest, 72 ± 1% in Amazon and 59 ± 7% in Cerrado. Although spatial patterns of litterfall were not significantly related to mean annual temperature (MAT) or mean annual precipitation (MAP), litterfall weakly increased with MAP (F_1,13_ = 3.03, *P* = 0.109; Fig. 3a), which explained 22% of its variance. A similar but stronger relationship between MAP and all plant components of litterfall (i.e. sum of leaves, twigs and reproductive parts; F_1,13_ = 5.36, *P* = 0.041) explained 33% of the variance (Fig. 3b). Litterfall variance was highest among biomes (30% of total variance), followed by across streams (23%), and lastly, within streams (11%; Table S4).

**Figure 4:**
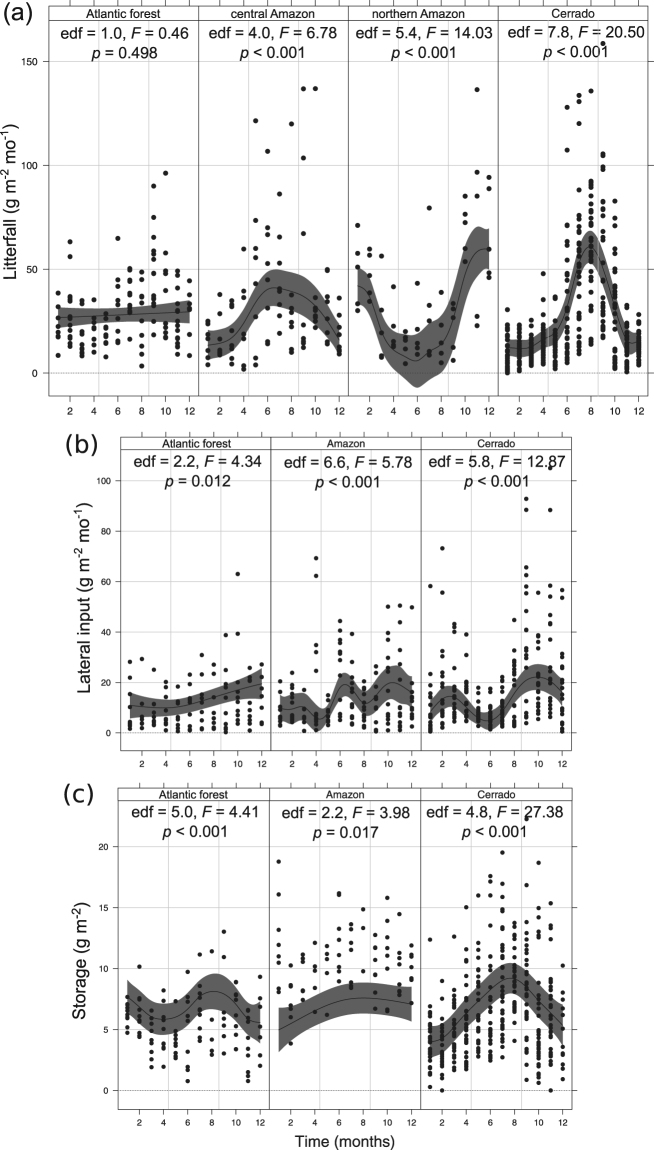
Temporal dynamics of **﻿(a)﻿**﻿ litterfall, **﻿(b)﻿**﻿ lateral inputs and **﻿(c)﻿** benthic storage (square-root transformed) over a year in each biome (Atlantic forest, Amazon and Cerrado) and the summary of additive mixed model results. Effective degrees of freedom (edf) of 1.0 represent a straight line (i.e., a linear pattern). Denominator degrees of freedom for *M*_*1Lf*_, *M*_*1Li*_ and *M*_*1St*_ are 694, 501 and 569, respectively. Black lines represent the smoothers of litterfall, lateral inputs and storage, and grey areas the 95% confidence intervals from models *M*_*1Lf*_, *M*_*1Li*_ and *M*_*1St*_, respectively. Litter inputs are in g per m^2^ per month and storage in g per m^2^.

**Figure 5:**
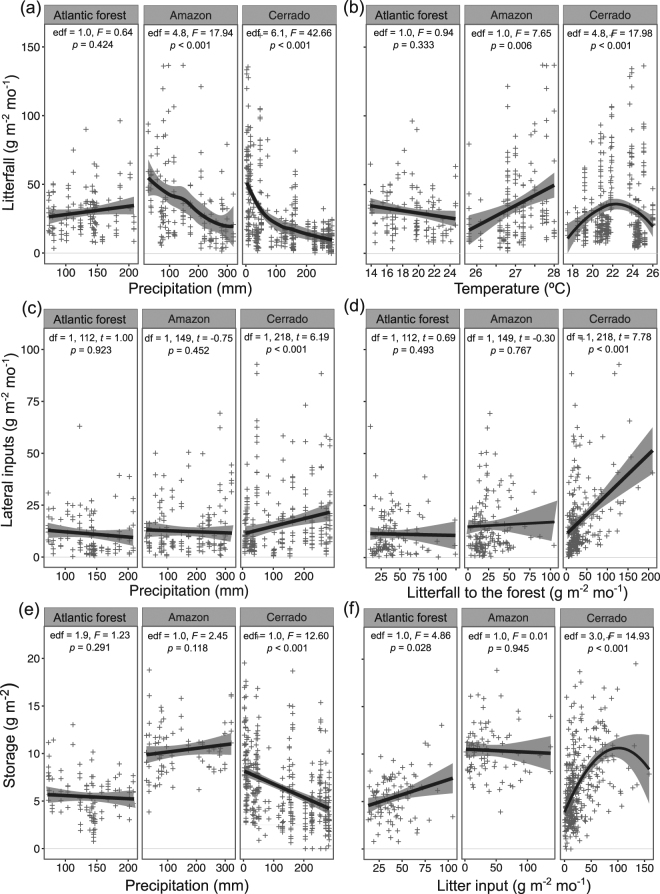
Relationship between litter inputs (g per m^2^ per month), storage (g per m^2^) and their temporal predictors in Atlantic forest, Amazon and Cerrado streams and the summary of mixed model results: (**a**) litterfall vs. precipitation; (**b**) litterfall vs. temperature; (**c**) lateral inputs vs. precipitation; (**d**) lateral inputs vs. litterfall to the forest; (**e**) storage vs. precipitation; and (**f**) storage vs. litter inputs. Degrees of freedom for smoother terms are estimates (those from models M_*2Lf*_ and M_*2St*_), and are represented by a straight line when equal to one (i.e. a linear effect). Denominator degrees of freedom for *M*_*2Lf*_ and *M*_*2St*_ are 694 and 553, respectively; and 6 for biome and 452 for the other terms for *M*_*2Li*_. Black lines represent the smoothers of litterfall, lateral inputs and storage, and grey areas the 95% confidence intervals from models *M*_*2Lf*_, *M*_*2Li*_ and *M*_*2St*_, respectively.

Temporal patterns of litterfall were consistently different among biomes, with lower variability over a year in Atlantic forest, intermediate in Amazon and higher in Cerrado (i.e., the higher degrees of freedom of additive mixed model, the higher the seasonality; Fig. 4): litterfall was constant throughout the year in Atlantic forest; peaked in June, July and August in central Amazon; between October to January in northern Amazon; and in July, August and September in Cerrado. Precipitation and temperature were important predictors of litterfall temporal dynamics, although effects were distinct among biomes: there was no relationship for Atlantic forest, a negative linear relationship between precipitation and litterfall for Amazon (both central and northern areas analyzed together) and a negative exponential relationship for Cerrado (Fig. 5a,b). In contrast, there was no relationship between temperature and litterfall for Atlantic forest, but a positive linear relationship for Amazon and a positive non-linear relationship for Cerrado (Fig. 5a,b).

### Lateral inputs

Lateral inputs were similar among Atlantic forest, Amazon and Cerrado (131 ± 25, 165 ± 7 and 213 ± 27 g leaf dry mass m^−2^ year^−1^; Table S3; Fig. S3). The contribution of lateral inputs to total litter inputs was 28 ± 13% for Atlantic forest, 28 ± 1% for Amazon and 41 ± 7% for Cerrado. Lateral inputs decreased as a function of precipitation in the driest month, and increased with the amount of total litterfall in the forest (F_2,6_ = 8.70; *P* = 0.017; Fig. 3c,d). These two predictors of spatial patterns of lateral inputs explained 66% of its variance. Lateral input variance was higher across streams (9%) than within streams (5%) or among biomes (<0.001%), although residual variance had the largest contribution (86%; Table S4).

Lateral inputs were more constant over a year in Atlantic forest, and more variable in Amazon and Cerrado (Fig. 4); increased from April (autumn) to December (late spring and early summer) in Atlantic forest; showed a bimodal trend with similar peaks in June and October–November in Amazon; and showed a bimodal trend in Cerrado but with a smaller peak in March (rainy season) and a larger one in October (beginning of rainy season and after litterfall peaks; Fig. 4). Precipitation and litterfall to the forest predicted temporal dynamics of lateral inputs , but significant interactions between precipitation and biome, and litterfall to forest and biome indicated significant positive relationships only for Cerrado (Fig. 5c,d).

### Storage

Litter storage was, on average, two times higher in Amazon than in Atlantic forest and three times higher than in Cerrado, but was similar between Atlantic forest and Cerrado (113 ± 1, 55 ± 5 and 38 ± 12 g leaf dry mass m^−2^; Table S3; Fig. S3). Storage increased as a function of MAP and stream depth, which explained 52% of its spatial pattern (F_2,8_ = 6.50; *P* = 0.021; Fig. 3e,f). Storage variance was higher among biomes than across or within streams (6% and <0.001%), but residual variance had the largest contribution (56%; Table S4).

Temporal dynamics of storage over the year was consistently distinct among biomes, with higher variability over a year in Atlantic forest and Cerrado and lower in Amazon (Fig. 4): storage showed a bimodal trend for Atlantic forest streams, with peaks in summer (beginning of the year) and winter (July to September); a peak from July to December in Amazon; and an evident peak from July to September (which correspond to the dry season) in Cerrado (Fig. 4). Precipitation and litter inputs were important predictors of temporal dynamics of storage, although effects were distinct among biomes: there was a negative linear relationship between precipitation and storage only for Cerrado streams, and positive relationships between litter input and storage for Atlantic forest (linear) and Cerrado (non-linear; Fig. 5e,f).

## Discussion

### Higher litterfall at Atlantic forest and Amazon as a result of higher precipitation

Allochthonous sources dominate energy flows in many tropical forested stream food webs^28^ as it occurs in streams of temperate zones^29^. Most of these allochthonous sources are represented by particulate organic matter in the form of leaf litter, which are of fundamental importance for stream food webs and ecosystem functioning^6^. However, to date there was no comprehensive study addressing how litter dynamics varies within the tropics or determining which are its environmental controls. Our study show how litter inputs and storage in tropical streams vary at multiple spatial scales within the tropics and which factors influence such variability, using a large-scale study involving streams across three tropical biomes.

We found that litterfall was higher in Amazon and Atlantic forest than in Cerrado and was positively related to precipitation, but not to temperature, partially supporting our prediction (Figs 1 and 2). These results contrast with those of another study^30^, which found no relationship between precipitation and annual litterfall in 81 South American tropical sites; however, 77 of those sites were in Amazon or Panamanian rainforests and none in Cerrado savanna, which occupies a large region in the center of South America^31^. It is thus likely that the spatial extent of our study (3 biomes and 30° of latitude) comprised a larger climatic gradient and also more varied forest types. Also, our findings indicated some similarities between tropical and temperate climates: temperate streams flowing through drier forests and with more seasonal precipitation regime (e.g., the Mediterranean biome) showed lower litter inputs than streams in Atlantic temperate forests, which have a more humid climate and more constant precipitation through the year^32^. The lack of a relationship between temperature and litterfall was unexpected, given the strong control that this climatic factor exerts on plant productivity globally^33^. Conversely, a pan-tropical analysis of net primary productivity – which is correlated with litterfall – found that temperature was the most important factor driving differences among tropical forest types^34^. The lack of a temperature effect in our study could be related to the distinct characteristics of the riparian forest compared to other types of forest. It is possible that riparian soil fertility played an important role in determining litterfall, as shown elsewhere ^20, 35, 36^, causing the differences observed among biomes. For example, the lowest litterfall production that we recorded, in riparian forests of Cerrado, may have been the result of its nutrient-poorer soils^37, 38^.

### Precipitation and temperature influence temporal dynamics of litterfall in Amazon and Cerrado

The negative relationship between litterfall and precipitation for Amazon and Cerrado indicate that precipitation is a limiting factor for litterfall regulation, supporting our prediction (Figs 1 and 2) and suggesting that litterfall helps plants reduce water stress during the driest periods^39, 40^. Higher litterfall in the driest months has been previously reported for riparian forests of Cerrado^13, 41^, in the Mediterranean climate^42^, and for tropical forests worldwide^19^, which contrast to the higher litterfall in autumn in temperate deciduous forests^43^. However, our study provides further evidence that this occurs in riparian forests of different tropical biomes and extends our understanding in important ways. Firstly, we found consistent evidence of litterfall seasonality in Amazon and Cerrado, and uniform litterfall rates over the year in Atlantic forest. These findings contradict the widespread perception of aseasonal litterfall in tropical riparian forests (mostly when climate is relatively constant year around)^44^ and evidence for different timing of litter inputs in different tropical riparian forests. Secondly, stronger litterfall seasonality in Cerrado and moderate l﻿itterfall seasonality in Amazon (both in central and northern areas) suggest important repercussions for litter decomposition and nutrient recycling in streams and riparian forests, as well as for aquatic and terrestrial food webs. This is due to the fact that leaf litter will not be supplied the same rates over the year, leading to probable reductions in litter quantity and changes in litter quality (i.e., chemical composition of stored litter in pools or soils due to biological or physical processes).

Also importantly, the uniform litterfall rates over the year observed in Atlantic forest may be the result of a mixture of subtropical Atlantic forest types (e.g., rain forests, Araucaria forest and semi-deciduous forest), which represents a mosaic of evergreen, semi-deciduous and deciduous trees^45^ that may sustain ‘constant’ litterfall rates over the year. Additionally, as the Atlantic forest biome is comprises by heterogeneous forest vegetation subtypes (e.g., rain, cloud, moist and dry forests in the coast and the interior areas) and our Atlantic forest sites were restricted to the southern portions of the Atlantic forest domain (mainly moist forests both in the coast and continental areas) our results for this biome should be interpreted with caution, mostly for different forest subtypes. The positive relationship between litterfall and temperature for Cerrado and Amazon indicates that temperature may also have an important effect on litterfall, as shown in other studies^46, 47^. Temperature increases evapotranspiration rates, which may lead to temporary water deficits that accelerate the abscission of senescent leaves^40^. Previous studies also suggested that light availability (e.g. solar radiation and day length) determines seasonal patterns in litterfall in tropical wet forests^48, 49^, because falling of mature leaves coincides with the appearance of new leaves during periods of higher radiation^50^. However, it is unlikely that light availability explains our seasonal pattern of litterfall in Cerrado, because periods of greatest day length occurred in different months or seasons at each site^51^; or the aseasonal pattern in Atlantic forest, where there was higher light availability during the summer (but see refs 51 and 52).

### Higher lateral inputs in more productive and drier riparian forests

In contrast to direct litterfall, litter coming from riparian soils may have undergone some degree of decomposition by physical or biological processes (depending on the time since litterfall) and may thus provide a different resource for stream food webs, because of leaching of labile compounds and microbial conditioning^53^. Thus, understanding the timing and magnitude of litter inputs from riparian soils represents an important step for future experimental or manipulative studies aiming to address their influence on stream ecosystem processes (e.g., litter decomposition, ecosystem metabolism and secondary production).

We found similar lateral inputs among Atlantic forest, Amazon and Cerrado streams, which did not support our prediction (Figs 1 and 2). However, as expected, we observed a positive relationship of lateral inputs with litterfall to the forest and a negative relationship with precipitation of the driest month. These findings suggest that higher lateral inputs occur in more productive riparian forests, because a higher amount of litter is available in riparian soils and is susceptible of reaching streams; and where drought periods are more intense and/or frequent, because dry litter is more easily transported^21, 54, 55^, although we found no relationship with wind frequency and bank slope. These discrepancies might be the result of interactions between wind, riparian density, ground complexity (i.e. plants, roots, dead trunks, rocks, etc.) and litter characteristics, understanding of which may require specific experimental studies^8^. Moreover, as many environmental factors can affect lateral litter transport, it is not surprising that a range of lateral litter contributions have been reported, from negligible amounts to even surpassing litterfall contributions (e.g., in mixed-hardwood forest^9^; in tropical rainforests^12^; in tropical savanna^13^; and in broadleaf forests^56^). These findings are supported by the higher variability of lateral litter inputs observed at smaller scales (86% of total at sampling sites or samplers), which suggest that local factors (e.g., riparian density, ground complexity, stream bank slope and litter characteristics) are more important than regional ones in driving its dynamics. Also, our results provide evidence that ignoring lateral inputs would result in considerable underestimation of total litter inputs to the stream, which according our data would be of 19–51% of total litter inputs to the stream.

### Temporal dynamics of lateral inputs depend on precipitation and soil litter accumulation in Cerrado

Lateral inputs and litterfall to the forest were positively related throughout the year only in Cerrado, indicating that lateral inputs were intensified in the most productive periods in this biome. Interestingly, lateral inputs increased with precipitation in Cerrado, contrary to our prediction, evidencing the higher lateral litter inputs mainly in the beginning of the rainy season. This is likely to occur through the mobilization of litter in the riparian floor by the wind during intense storms, which although sporadic are more common to occur in the dry-wet transition. In contrast, there was no temporal relationship between lateral inputs and litterfall to the forest or precipitation in Amazon or Atlantic forest, suggesting that litter transport in these biomes is not intensified by litter accumulation in riparian soils or overland flow, which is expected to be of minor importance on the well drained soils of riparian zones studied. The lack of relationship between lateral inputs and litterfall to the forest is striking and might indicate the lower movement of litter in riparian soils of Amazon and Atlantic forest, probably slowed down by the high humidity in most periods of the year. Previous studies have reported either a positive or no relationship between precipitation and lateral litter transport^55, 57, 58^, reflecting regional patterns and suggesting that direct field measures (e.g., overland flow and wind intensity on the floor base) should provide a better representation of a highly local variable processes such as litter transport in riparian soils.

### Litter storage increases with annual precipitation and stream depth

Benthic litter storage is a major energy source for secondary production in forest stream food webs^6, 7^, influencing nutrient cycles and the export of particulate and dissolved organic carbon^59^. Benthic litter also helps with channel stability (through reducing bank erosion), increases stream retentiveness^60^ and it is habitat for microorganisms, invertebrates and fishes^61^. Thus, spatial and temporal dynamics of litter storage potentially have important consequences for all the above processes and organisms.

Our results showed storage to increase with annual precipitation and water depth. Similarly, Jones^62^ found that litter storage was directly related to annual precipitation, suggesting that storage increased as a result of enhanced litter production with precipitation. The positive relationship between storage and water depth was contrary to our predictions but might be related to the higher litter accumulation in pools, which are deeper and in consequence low-flow habitats that are able to store larger amounts of materials than riffle habitats. The lack of a relationship with litter inputs suggests that annual storage in these streams is primarily driven by their low retention capacity (5 to 19% of litter inputs) and high downstream litter export in relation to litter inputs. This result contrasts with Jones^62^, who demonstrated an increase of litter storage with inputs in North American streams, but is in accordance to another study in Neotropical streams where low storage (~10% of total inputs; 13–153 g leaf dry mass m^−2^) was also reported despite high litter inputs (590–918 g leaf dry mass m^−2^ y^−1^)^15^. In our study, storage was up to 3 times higher in Amazon than in other biomes, which is surprising because Amazon streams had sand substrates, which generally show lower retention than cobble-dominated streams^62^. Also, the high variance (ca. 40%) of litter storage among biomes and its relation with annual precipitation suggest that a considerable proportion of storage dynamics was by regional processes that could directly influence litter retention and export (e.g. precipitation regime and hydrology). Taken together, these results suggest that spatial pattern in litter storage is partly due to biome type, despite large unexplained variance.

### Temporal dynamics of litter storage are driven by precipitation and litter inputs

We observed distinct temporal patterns of litter storage among biomes, which were driven by precipitation and litter inputs in Cerrado and inputs in Atlantic forest, supporting our prediction. This indicates that temporal patterns of in-stream storage in Cerrado are more predictable, given that higher inputs coincide with base-flow conditions (during the dry season). Also, temporal storage patterns of Cerrado demonstrated a massive accumulation of benthic litter until the rainy season starts, when the beginning of rainy season flushed out the system most of benthic litter to downstream, banks or hyporheic zone. Notably, most of the removed litter might be in the initial stages of decomposition, given the low decomposition rates reported for Cerrado streams (~20–50% mass loss in 75–120 days^63, 64^). It is possible that storage in Atlantic forest is only predicted by litter inputs due to well distributed precipitation throughout the year, which can limit litter accumulation in streams through the occurrence of spates which scoured benthic litter (which were not reflected in monthly precipitation). This empirical evidence supports theoretical predictions of the role of hydrological regimes in litter availability in streams^65^ and suggests that retained litter is transported downstream before it is processed by biological communities.

In contrast to Atlantic forest and Cerrado, Amazon streams were characterized by high litter storage throughout the year (Fig. 4), and a lack of a relationship with precipitation. For instance, the annual range of litter storage in Amazon streams (43–210 g leaf dry mass m^−2^) was higher than those of Atlantic forest and Cerrado streams (4–144 and 5–172 g leaf dry mass m^−2^, respectively), which were similar or even higher than those observed for temperate deciduous forest streams (e.g., 5–40^66^, 0–20^67^, 0–78^68^ g leaf dry mass m^−2^). These results suggest that Amazon streams did not experience large or periodic litter export to downstream reaches over the year, unlike Cerrado and Atlantic forest streams, respectively. This can be the result of topographic and hydrological characteristics of Amazon streams draining *terra firme* forests, where the altitudinal gradient is low (60–100 m asl) and high precipitation events usually do not disturb the streambed^69, 70^. This finding indicates that most benthic litter in Amazon streams might have enough time to be colonized by microbial and invertebrate communities, and possibly its decomposition is driven by different agents and routes than in Atlantic forest streams.

## Conclusions

Our study provides comprehensive evidence of the spatial patterns and temporal dynamics of litter inputs and storage, and the major factors influencing them, in tropical streams across several biomes. Firstly, higher litter inputs occurred in the most humid biomes (Atlantic forest and Amazon forest) because of a positive effect of precipitation on plant production. Secondly, higher litter storage was observed in Amazon forest than in Atlantic forest or Cerrado savanna streams, in relation to higher annual precipitation and/or higher water stream depth. Thirdly, there were distinct temporal patterns of litter inputs and storage according to the type of biome: uniform litter inputs but rather seasonal storage in Atlantic forest, and seasonal inputs in both Amazon forest and Cerrado savanna, but aseasonal litter storage in Amazon forest. Fourthly, temporal patterns of inputs were mostly driven by precipitation (although temperature and litter availability were also important), while storage was determined by litter inputs and precipitation. In conclusion, these results evidence that major differences in plant litter dynamics in streams across tropical biomes are mostly influenced by precipitation. However, we still know remarkably little about how this variability might affect litter decomposition, energy flow and complex food webs in streams ecosystems at regional or at broad scales (e.g., Parton, *et al*.^71^, Boyero, *et al*.^72^, Boyero, *et al*.^73^). This information is crucial to predict changes in stream ecosystem functioning and potential effects on the global carbon cycle as a result of future changes in temperature and precipitation regimes^74^.

## Methods

### Study sites

**Figure 6:**
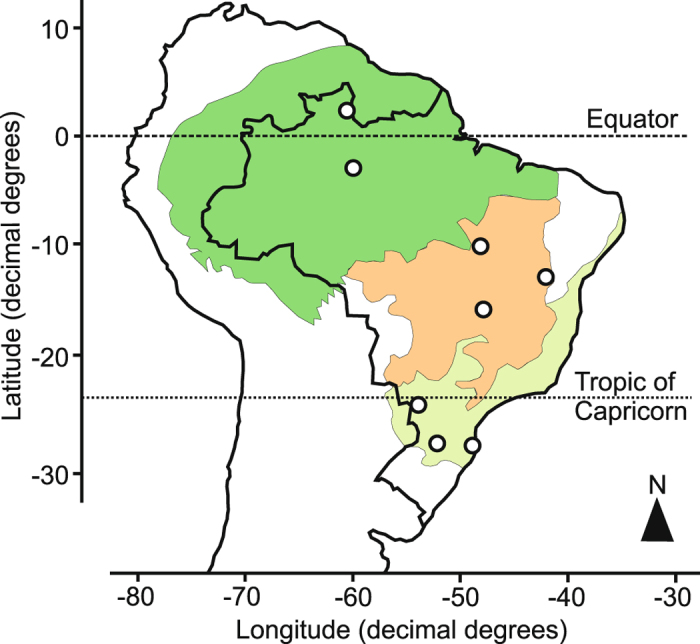
Location of study sites in Atlantic Forest (light green area), Cerrado savanna (orange area) and Amazon forest (dark green area) biomes. This figure was generated using ‘ggmap’ package (http://journal.r-project.org/archive/2013-1/kahle-wickhampdf) in R (version 3.2.2; https://www.R-project.org/).

Our study was conducted in 13 streams located in 3 biomes in Brazil: the subtropical Atlantic forest (3 streams), the Amazon tropical forest (3) and the Cerrado tropical savanna (7). Study sites were located at latitudes ranging from 2°N to 28°S (Fig. 6, Table S1). We selected 1^st^–3^rd^ order streams < 5 m wide and < 50 cm deep (estimated at low flow conditions), with dense riparian canopy (> 70%), in watersheds with no apparent anthropogenic impacts. The riparian forests in all three biomes were highly species diverse, containing deciduous, semi-deciduous and evergreen species (> 50–122 species in Atlantic forest, > 50–62 in Amazon and 29–112 in Cerrado; Table S2). Atlantic forest streams were located in the interior (2 streams) and coast (1) areas of Brazil; the climate is subtropical with frequent precipitation and no dry season; vegetation is mainly composed of Araucaria rainforest and semi-deciduous forest. Cerrado savanna streams drain through dense corridors of evergreen forest known as gallery forest^35^ and experience a tropical seasonal climate with a dry season from May through September that coincides with the coldest months of the year. The Amazon biome encompasses the largest tropical rainforest in the world; our streams drained non-flooded (*terra firme*) forests located in the central (2 streams) and northern Amazon (1); the climate is tropical humid, with central Amazon sites characterized by a rainy season from December through May and a modest dry season from June through November, and northern Amazon sites with a rainy season from April to September and a pronounced dry season from October to March.

### Experimental design and procedure

In each stream, we conducted the experiment at 5 equally distanced sampling sites within a 50–100 m long reach. Litterfall and lateral litter inputs were estimated using suspended and lateral traps, respectively. Suspended traps consisted of 90 plastic buckets (18 per site) placed 2 m above the streambed, with a 26-cm diameter and small holes on the bottom to allow water to drain; their total sampling area was 4.75 m^2^. Lateral collectors consisted of 20 traps (4 per site) of 50 × 25 × 50 cm and made of 1-mm mesh; they were distributed along the stream bank and fixed to the soil. Additionally, we estimated litterfall to the riparian forest floor with 10 suspended nets (2 per site) of 1-m^2^ area and 1-mm mesh. Benthic litter storage was estimated with 15 Surber samples (3 per site taken randomly, including pool and riffle areas) of 0.10 m^2^ and 250-μm mesh that were further sieved through a 1-mm mesh.

Samples were collected once a month for a year (Fig. S1). They were transported to the laboratory, oven dried and sorted into four categories: leaf litter, twigs, reproductive parts (fruits, flowers and seeds) and unidentified parts. However, we mostly focused on leaf litter (henceforth “litter”) in further analyses because it represented the majority of total particulate organic inputs (>60% of dry mass [DM]; SI 2), while the other fractions were absent in many sites and showed large variance across replicates and over time. Monthly litterfall and lateral inputs were estimated as litter DM per m^2^ per year at each sampling site. Storage was estimated as litter DM per m^2^ on each occasion.

At each site we estimated a set of variables related to spatial patterns of litterfall, lateral inputs and storage: stream and bank slope (with a clinometer), and water depth and width (cross sections with 5 depth measures each). We calculated the coefficient of variation (CV) of the width/depth ratio of each site as a measure of channel heterogeneity (as an indicator of stream retentiveness). For each of these variables, we used the 5 values from the different sites to calculate a mean value per stream. Additionally, we extracted temperature and precipitation data for each stream from the WorldClim database v.1.3^75^ at the highest resolution (2.5 min of arc) using DIVA-GIS software, 7.5.0.0 (http://www.diva-gis.org), and wind frequency from the National Institute of Meteorology of Brazil (Automatic Stations from http://www.inmet.gov.br). We used the average of minimum and maximum temperatures for each month to calculate monthly mean temperature, which was used for temporal analyses. For spatial analyses, we used the following climatic predictors: mean annual precipitation (MAP), mean annual temperature (MAT), precipitation of the driest month (PDM, as an indicator of the presence of dry periods) and wind frequency.

### Data analysis

#### Spatial Models

We explored the relationships between litterfall, lateral inputs, storage and their environmental predictors with linear models, after averaging monthly measurements and site data within a stream. Litterfall predictors included MAP and MAT; lateral input predictors were litterfall to the forest (as a surrogate of fresh litter availability in forest soils), wind frequency, PDM and bank slope; and storage predictors were MAP, litter inputs (sum of litterfall and lateral inputs), stream slope, water depth and channel heterogeneity. We first used the variance inflation factor and a cut-off value of 3 to remove collinear explanatory variables^32^. Next, we selected the best models by removing any non-significant variables and assessing model improvements based on the Akaike Information Criterion (AIC) (Table S2). Models were fitted using the ‘stats’ package and plots were drawn with the ‘ggplot2’ package^76^ (and in association with ‘ggmap’ package in the case of Fig. 6) in R^77^; version 3.2.2.

#### Temporal Models

We examined temporal dynamics of litterfall, lateral inputs and storage, as well as the effects of environmental factors, with additive mixed models (GAMM) using a normal distribution and the identity-link function^78, 79^. We used this type of model instead of a linear model because scatterplots of litter inputs and storage (on the y-axis) for each biome, with the covariates (time, precipitation, temperature and litterfall to the forest) on the x-axis, showed clear non-linear patterns^79, 80^. Importantly, additive models (also called smoothing models) allow for non-linear relationships between the response variable and multiple explanatory variables, in contrast to linear models^81^. Also, the amount of smoothing in an additive model is expressed as effective degrees of freedom (edf) for a smoother. Thus, the higher the edf, the lower the linearity of a curve^79^. Initial data exploration using Cleveland dot- and boxplots revealed outliers in the storage data, which required square-root transformation prior to analysis. Examination of multi-panel scatterplots indicated contrasting patterns of litterfall within the Amazon biome, so this biome was separated into central and northern Amazon, but only for litterfall comparisons. All models were fitted using the ‘mgcv’^82^ and ‘nlme’^83^ packages in R.

We firstly fitted a model to describe temporal patterns for each response variable (litterfall, lateral inputs and storage) that excluded the environmental factors. The explanatory variables in this model were biome (Atlantic forest, Amazon or Cerrado), time (number of the month within a year; continuous variable) and the interaction between biome (categorical) and time (fitted as a smoother). Secondly, we fitted a model that included the environmental covariates. For litterfall, the explanatory variables were precipitation (as a surrogate for flow; smoother), temperature (continuous variable) and the interaction between precipitation and biome. The lateral input model was first fitted using an additive mixed model, with precipitation and litterfall to the forest as smoothers. However, effective degrees of freedom for these smoothers were 1, indicating a linear effect, so a linear mixed model was more appropriate. Explanatory variables for lateral inputs were precipitation (continuous variable), litterfall to forest (continuous variable) and their interaction with biome. For litter storage, the explanatory variables were precipitation, litterfall to the stream and their interaction with biome (see full models in SI 2). The interactions in additive mixed models were fitted using the ‘by’ command in the ‘mgcv’ package in R. Cross-validation was used to estimate the optimal amount of smoothing^78^.

We extracted variance components and standard deviations of litterfall, lateral inputs and storage for each hierarchical scale: biomes, streams nested within biomes (hereafter ‘across streams’) and sites nested within streams (hereafter ‘within streams’) using the ‘VarCorr’ function in linear mixed effects models. Biome was treated as a random factor purely to allow comparison with other components^84^.

### Data Availability

The datasets generated during and/or analysed during the current study are available in the Open Science Framework repository using the link: https://osf.io/2rh5u/?view_only=5f8d88a4ea7a400aad82cb769209d5c1.

## Electronic supplementary material

Supplementary Information
