# Supplementary material for: Plant litter dynamics in the forest-stream interface: precipitation is a major control across tropical biomes
Source: Sci Rep. 2017 Sep 7;7:10799. doi: 10.1038/s41598-017-10576-8 (PMC5589825; doi:10.1038/s41598-017-10576-8)
Supplement: Supplementary file 1 — Supplementary Information [file 41598_2017_10576_MOESM1_ESM.doc]

Supporting Information

Title: **Plant litter dynamics in the forest-stream interface: precipitation is a major control across tropical biomes**

Alan Mosele Tonin^1,2*^, José F. Gonçalves Junior^1^, Paulino Bambi^1^, Sheyla R. M. Couceiro^3^, Lorrane A. M. Feitoza^4^, Lucas E. Fontana^5^, Neusa Hamada^6^, Luiz U. Hepp^5^, Vânia G. L. Kowalczuk^4^, Gustavo F. M. Leite^1^, Aurea L. Lemes-Silva^7^, Leonardo K. Lisboa^7^, Rafael C. Loureiro^5^, Renato T. Martins^6^, Adriana O. Medeiros^8^, Paula B. Morais^9^, Yara Moretto^10^, Patrícia A. Oliveria^6^, Evelyn B. Pereira^6^, Lidiane F. Pereira^4^, Javier Pérez^2^, Mauricio M. Petrucio^7^, Deusiano F. Reis^9^, Renan Rezende^1^, Nadia Roque^8^, Luiz E. P. Santos^4^, Ana E. Siegloch^7^, Gabriela Tonello^5^ and Luz Boyero^2, 11, 12^

^1^Limnology Lab, Dept. of Ecology, IB, Univ. of Brasília (UnB), 70910-900, Brasília, DF, Brazil.

^2^Faculty of Science and Technology, Univ. of the Basque Country (UPV/EHU), Leioa, Spain

^3^Lab de Ecologia e Taxonomia de Invertebrados, Univ. Federal do Oeste do Pará (UFOPA), Inst. de Ciências e Tecnologia das Águas, Campus Amazônia Boulevard, 68040-470, Santarém, PA, Brazil.

^4^Univ. Federal de Roraima, Centro de Estudos da Biodiversidade, 69304-000, Boa Vista, RR, Brazil.

^5^Univ. Regional Integrada do Alto Uruguai e das Missões, Dept. Ciências Biológicas, 99709-910, Erechim, RS, Brazil.

^6^Coordenação de Biodiversidade, Instituto Nacional de Pesquisas da Amazônia (INPA), 69067-375, Manaus, AM, Brazil

^7^Lab. de Ecologia de Águas Continentais, Dept. de Ecologia e Zoologia, Centro de Ciências Biológicas, Univ. Federal de Santa Catarina, 88040-900, Florianópolis, SC, Brazil.

^8^Univ. Federal da Bahia, Dept. de Botânica, Inst. de Biologia, 40170-115, Salvador, BA, Brazil.

^9^Lab. Microbiologia Ambiental e Biotecnologia, Univ. Federal do Tocantins, 77001-923 Palmas, TO, Brazil.

^10^Univ. Federal do Paraná, Dept. de Biodiversidade, 85950-000, Palotina, PR, Brazil.

^11^IKERBASQUE, Basque Foundation for Science, Bilbao, Spain.

^12^College of Marine and Environmental Sciences and TropWater, James Cook Univ., Townsville, QLD 4811, Australia.

*Corresponding author: Alan M. Tonin. Email: tonin.alan@gmail.com. Telephone: +61 31072986. Fax: +61 32721497

SI 1 Information of sites and sampling period

**Table S1.** Location of study streams per biome (AF, Atlantic forest; CE, Cerrado savanna; AM, Amazon forest), code of streams, latitude (Lat) and longitude (Long; in degrees), altitude (Alt; m asl), MAT (mean annual precipitation; ºC), TS (temperature seasonality; standard deviation of monthly mean temperature × 100), MAP (mean annual precipitation; mm), PS (precipitation seasonality; coefficient of variation of monthly mean precipitation), PDM (precipitation of the driest month; mm), dominant substrate type, stream depth (m) and wetted width (m), canopy cover of streambed (%), and slope of bank and channel (in degrees). Stream depth and wetted width refer to the base-flow conditions. Depth, width, canopy cover, bank slope and channel slope are means of five sites per stream (see methods for additional details).

| Biome | Code | Lat | Long | Alt | MAT | TS | MAP | PS | PDM | Substrate | Depth | Width | Canopy cover | Bank slope | Channel slope |
| --- | --- | --- | --- | --- | --- | --- | --- | --- | --- | --- | --- | --- | --- | --- | --- |
| AF | CGRANDE | -27.7 | -48.5 | 79 | 19.6 | 287 | 1427 | 37 | 73 | boulder | 0.22 | 4.6 | 80 | 28 | 26 |
| AF | GAUR | -27.6 | -52.1 | 574 | 18 | 312 | 1823 | 15 | 124 | cobble | 0.13 | 3.0 | 69 | 19 | 10 |
| AF | QUATI | -24.3 | -53.9 | 295 | 20.9 | 317 | 1524 | 26 | 74 | silt | 0.40 | 2.5 | 84 | 4 | 2 |
| CE | CAPET | -16.0 | -47.9 | 1090 | 20.7 | 112 | 1650 | 80 | 8 | gravel | 0.23 | 2.9 | 84 | 23 | 5 |
| CE | CVEADO | -15.9 | -47.8 | 1079 | 20.7 | 112 | 1650 | 80 | 8 | cobble | 0.23 | 2.8 | 87 | 5 | 3 |
| CE | RONCAD | -15.9 | -47.9 | 1069 | 20.7 | 112 | 1650 | 80 | 8 | silt | 0.35 | 3.0 | 92 | 2 | 2 |
| CE | BOIAD | -13.0 | -41.3 | 984 | 19.9 | 130 | 918 | 59 | 22 | sand | 0.62 | 1.8 | 75 | 5 | 1 |
| CE | BURIT | -10.3 | -48.1 | 629 | 24.6 | 61 | 1730 | 80 | 3 | sand | 0.36 | 1.9 | 86 | 39 | 1 |
| CE | BVISTA | -10.3 | -48.2 | 643 | 24.6 | 61 | 1730 | 80 | 3 | gravel | 0.10 | 1.5 | 93 | 33 | 2 |
| CE | SBENTO | -10.3 | -48.1 | 544 | 24.6 | 61 | 1730 | 80 | 3 | sand | 0.54 | 1.7 | 93 | 26 | 1 |
| AM | ACARA | -3.0 | -60.0 | 82 | 27.1 | 49 | 2193 | 42 | 77 | sand | 0.30 | 2 | 86 | 3 | 5 |
| AM | BBRANCO | -2.9 | -59.9 | 98 | 27.1 | 49 | 2193 | 42 | 77 | sand | 0.62 | 1.8 | 85 | 3 | 2 |
| AM | ASERRA | 2.4 | -60.6 | 100 | 26.8 | 63 | 1646 | 84 | 26 | sand | 0.17 | 4.2 | 79 | 5 | 4 |

**Table S2.** Plant diversity (number of species, including trees and lianas) in the riparian forest of study streams per biome (AF, Atlantic forest; CE, Cerrado savanna; AM, Amazon forest). Local surveys of plant diversity were performed using 10 plots (10×10m) along the watercourse (see more details in Bambi et al. 2017). Local estimations were performed through visual estimates of plant diversity by botanists.

| Biome | Code | Plant diversity | Source |
| --- | --- | --- | --- |
| AF | CGRANDE | 122 | Lisboa et al. 2015[^1^](#_ENREF_1) |
| AF | GAUR | 80 | Capellesso et al. 2016[^2^](#_ENREF_2) |
| AF | QUATI | > 50 | Local estimation |
| CE | CAPET | 70 | Bambi et al. 2017[^3^](#_ENREF_3) |
| CE | CVEADO | 112 | Bambi et al. 2017[^3^](#_ENREF_3) |
| CE | RONCAD | 29 | Bambi et al. 2017[^3^](#_ENREF_3) |
| CE | BOIAD | 51 | Local survey |
| CE | BURIT | 87 | Local survey |
| CE | BVISTA | 83 | Local survey |
| CE | SBENTO | > 80 | Local estimation |
| AM | ACARA | 58 | Local survey |
| AM | BBRANCO | 62 | Local survey |
| AM | ASERRA | > 50 | Local estimation |


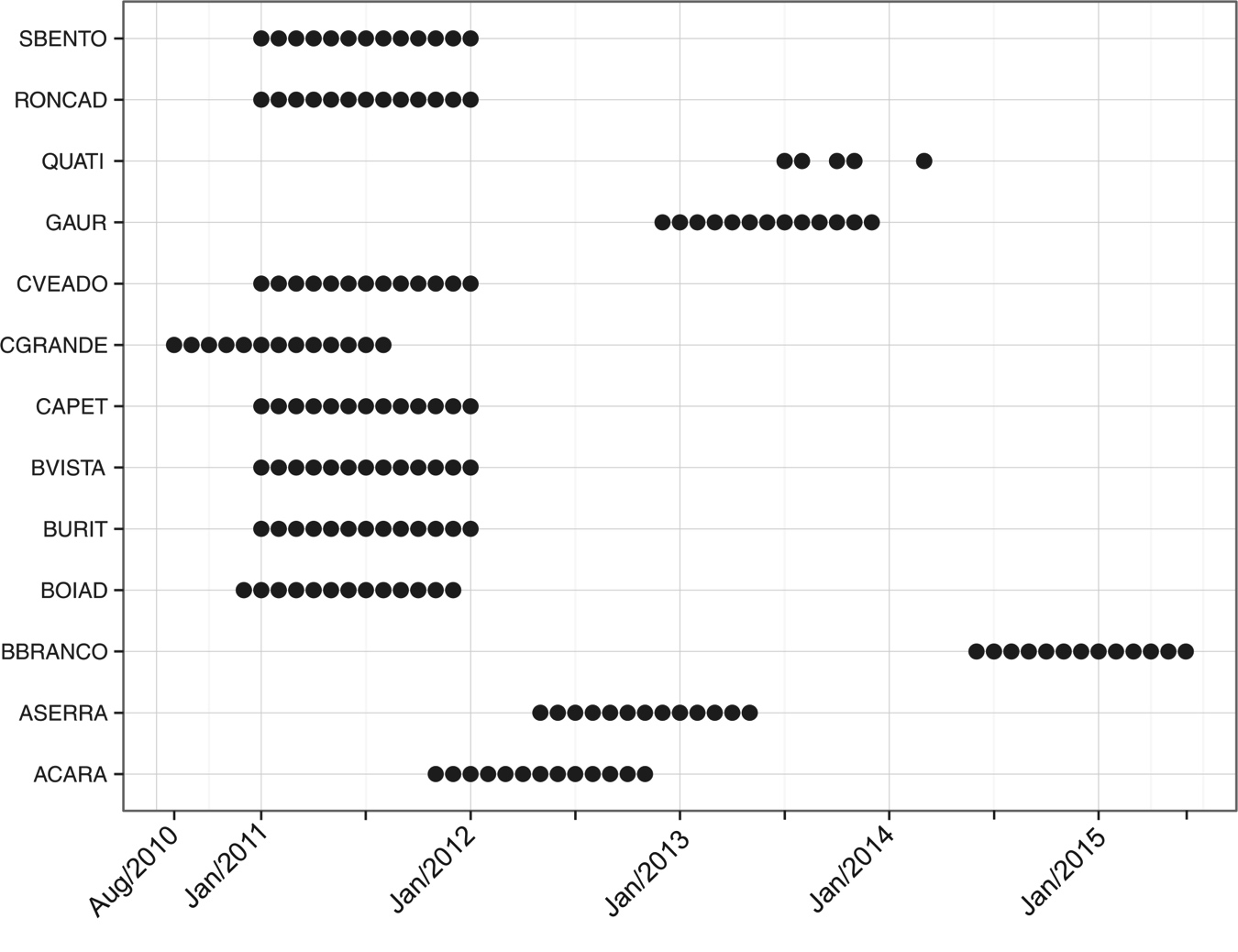


**Figure S1.** Interval of sampling at each stream (codes are presented in Table 1). The first circle of each stream represent when the samplers were installed in the field.

SI 2 Temporal models

Model *M_1_* describe temporal patterns for each response variable (litterfall, *M_1Lf_;* lateral inputs, *M_1Li_*; and storage, *M_1St_)*, which excluded the environmental factors. The explanatory variables in this model were biome (Atlantic forest, Amazon or Cerrado; categorical variable), time (number of the month within a year; continuous variable) and the interaction between biome and time (fitted as a smoother). Model *M_2_* included the environmental covariates: precipitation (PREC), temperature (TEMP), litterfall to forest (LF; continuous variable) and litterfall to the stream (LS), with respect to each response variable.

*M_1Lf_, M_1Li_*, *M_1St_*: Litter inputs or storage*_ijk_* = α + *f* (time*_i_*) × biome*_ijk_* + a*_k_* + a*_j|k_* + ε*_ijk_*

*M_2Lf_*: Litterfall*_ijk_* = α + *f* (PREC*_i_*) × biome*_ijk_* + *f* (TEMP*_i_*) × biome*_ijk_* + a*_k_* + a_j|k_ + ε*_ijk_*

*M_2Li_*: Lateral Inputs*_ijk_* = α + PREC*_ijk_* × biome*_ijk_* + LF*_ijk_* × biome*_ijk_* + a*_k_* + a_j|k_ + ε*_ijk_*

*M_2St_*: Storage*_ijk_* = α + *f* (PREC*_i_*) × biome*_ijk_* + *f* (LS*_i_*): biome*_ijk_* + a*_k_* + a_j|k_ + ε*_ijk_*,

where α is an intercept; *f* is the smoothing function; a*_k_* and a_j|k_ are random intercepts allowing for variation between the streams and between samples within the same stream, respectively; and ε is independently, normally distributed error with mean zero and variance σ^2^.

Temporal autocorrelation between subsequent samplings was examined using the autocorrelation function of the ‘nlme’ package with respect to month. Temporal autocorrelation was detected in litterfall data and therefore we used an auto-regressive model of order 1. Spatial autocorrelation was detected for litter inputs and storage data with variograms of normalized residuals of each model. To incorporate spatial dependency of data into models, sampling sites nested within streams were considered as random components. Visual inspection of residuals plots and initial data exploration indicated violation of homogeneity in most cases, requiring the use of a variance structure that allows for different residual spread within biomes over time ([i.e., 'VarIdent’ functi^on4^](#_ENREF_4)). The optimal random structure was defined selecting models with the lowest AIC. Once the optimal random structure was found, we selected the best model in terms of fixed structure by removing any non-significant variables or interactions.

SI 3 Supplementary results

**Table S3.** Summary of backward model selection based on Akaike information criterion (AIC) for litterfall, total litterfall (sum of all litter categories), lateral inputs and storage in streams. The *p*-value refers to the comparison between 1^st^ and 2^nd^, 2^nd^ and 3^rd^ model, and so on; and non-significant *p*-values indicate that both models are similar (at 5% level). MAT, mean annual temperature; MAP, mean annual precipitation; PS, precipitation seasonality; WF, wind frequency; PDM, precipitation of the driest month; SLOPE, bank and stream slope for lateral input and storage models, respectively; LI, litter inputs; DEPTH, stream depth; HCM, heterogeneity of channel morphology.

|  | **Model** | **DF** | **AIC** | ***p*** |
| --- | --- | --- | --- | --- |
|  | **Litterfall** |  |  |  |
| 1 | MAT + MAP + PS | 5 | 154.7 |  |
| 2 | MAP + PS | 4 | 153.2 | 0.514 |
| 3 | MAP | 3 | 152.4 | 0.267 |
|  | **Total litterfall** |  |  |  |
| 1 | MAT + MAP + PS | 5 | 163.4 |  |
| 2 | MAP + PS | 4 | 161.4 | 0.803 |
| 3 | MAP | 3 | 160.3 | 0.342 |
|  | **Lateral inputs** |  |  |  |
| 1 | LF + WF + PDM + SLOPE | 6 | 94.6 |  |
| 2 | LF + WF + PDM | 5 | 93.0 | 0.395 |
| 3 | LF + PDM | 4 | 91.8 | 0.795 |
|  | **Storage** |  |  |  |
| 1 | LI + MAP + SLOPE + DEPTH + HCM | 7 | 104.2 |  |
| 2 | MAP + SLOPE + DEPTH + HCM | 6 | 102.2 | 0.847 |
| 3 | MAP + DEPTH + HCM | 5 | 102.0 | 0.181 |
| 2 | MAP + DEPTH | 4 | 102.1 | 0.138 |

**Fractions of litter inputs**: Litterfall was, on average ± SE, 70 ± 2% of leaves, 13 ± 2% of twigs, 9 ± 2% of reproductive parts and 8 ± 1% of other. Lateral litter inputs were 57 ± 5% of leaves, 19 ± 4% of twigs, 9 ± 3% of reproductive parts, and 15 ± 4% of other litter types. Benthic storage was 47 ± 5% of leaves, 24 ± 5% of twigs, 15 ± 7% of reproductive parts and 14 ± 3% of others (Fig. S3).

**Table S4.** Summary of linear mixed effects models testing for differences in monthly litterfall, lateral inputs and storage among Atlantic forest (AF), Amazon forest (AM) and Cerrado savanna (CE) biomes. AF was used as a baseline (intercept) for comparisons with AM and CE, and AM vs. CE comparison was obtained reordering the dataset.

|  | Value | SE | df | *t* | *P* |
| --- | --- | --- | --- | --- | --- |
| *Litterfall* |  |  |  |  |  |
| Intercept | 27.22 | 4.10 | 631 | 6.64 | < 0.001 |
| AM vs AF | 0.69 | 6.36 | 10 | 0.11 | 0.916 |
| CE vs AF | -16.95 | 4.80 | 10 | -3.53 | 0.005 |
| AM vs CE |  | 5.47 | 10 | 3.23 | 0.009 |
| *Lateral Inputs* |  |  |  |  |  |
| Intercept | 10.05 | 2.48 | 458 | 4.05 | < 0.001 |
| AM vs AF | -3.00 | 3.17 | 6 | -0.95 | 0.381 |
| CE vs AF | -1.34 | 3.02 | 6 | -0.44 | 0.673 |
| AM vs CE |  | 2.62 | 6 | -0.63 | 0.551 |
| *Storage* |  |  |  |  |  |
| Intercept | 24.4 | 7.0 | 517 | 3.5 | < 0.001 |
| AM vs AF | 74.4 | 11.3 | 8 | 6.6 | < 0.001 |
| CE vs AF | 7.8 | 8.3 | 8 | 0.9 | 0.375 |
| AM vs CE |  | 9.9 | 8 | 6.7 | < 0.001 |


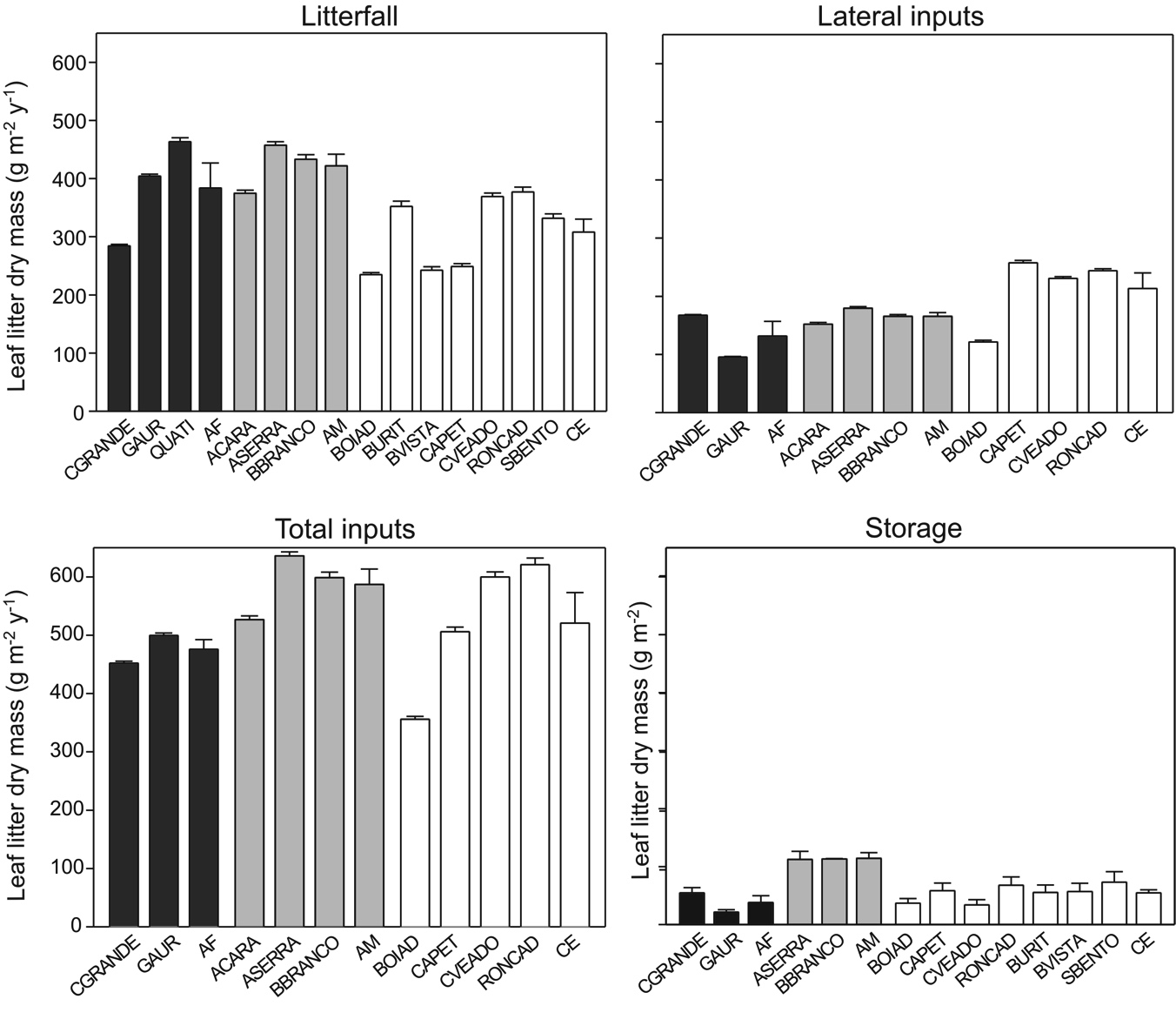


**Figure S2.** Annual estimates (mean ± SE) of litterfall, lateral inputs and storage at Atlantic Forest (AF; black bars), Amazon (AM; grey bars) and Cerrado (CE; white bars) biomes.

**Table S5.** Estimated variance, standard deviation (SD) and percent of total variance of litterfall, lateral inputs and benthic storage partitioned in spatial scales (among biomes, across streams and within streams) from the linear mixed effects model.

| Terms | Variance | SD | % total variance |
| --- | --- | --- | --- |
| *Litterfall* |  |  |  |
| Biome | 61.4 | 7.8 | 30 |
| Across streams | 46.5 | 6.8 | 23 |
| Within streams | 22.1 | 4.7 | 11 |
| Residuals | 72.4 | 8.5 | 36 |
| *Lateral Inputs* |  |  |  |
| Biome | < 0.001 | < 0.01 | < 0.001 |
| Across streams | 8.68 | 2.95 | 9 |
| Within streams | 5.46 | 2.34 | 5 |
| Residuals | 89.27 | 9.45 | 86 |
| *Storage* |  |  |  |
| Biome | 6.43 | 2.53 | 38 |
| Across streams | 0.96 | 0.98 | 6 |
| Within streams | < 0.001 | < 0.001 | < 0.001 |
| Residuals | 9.31 | 3.05 | 56 |


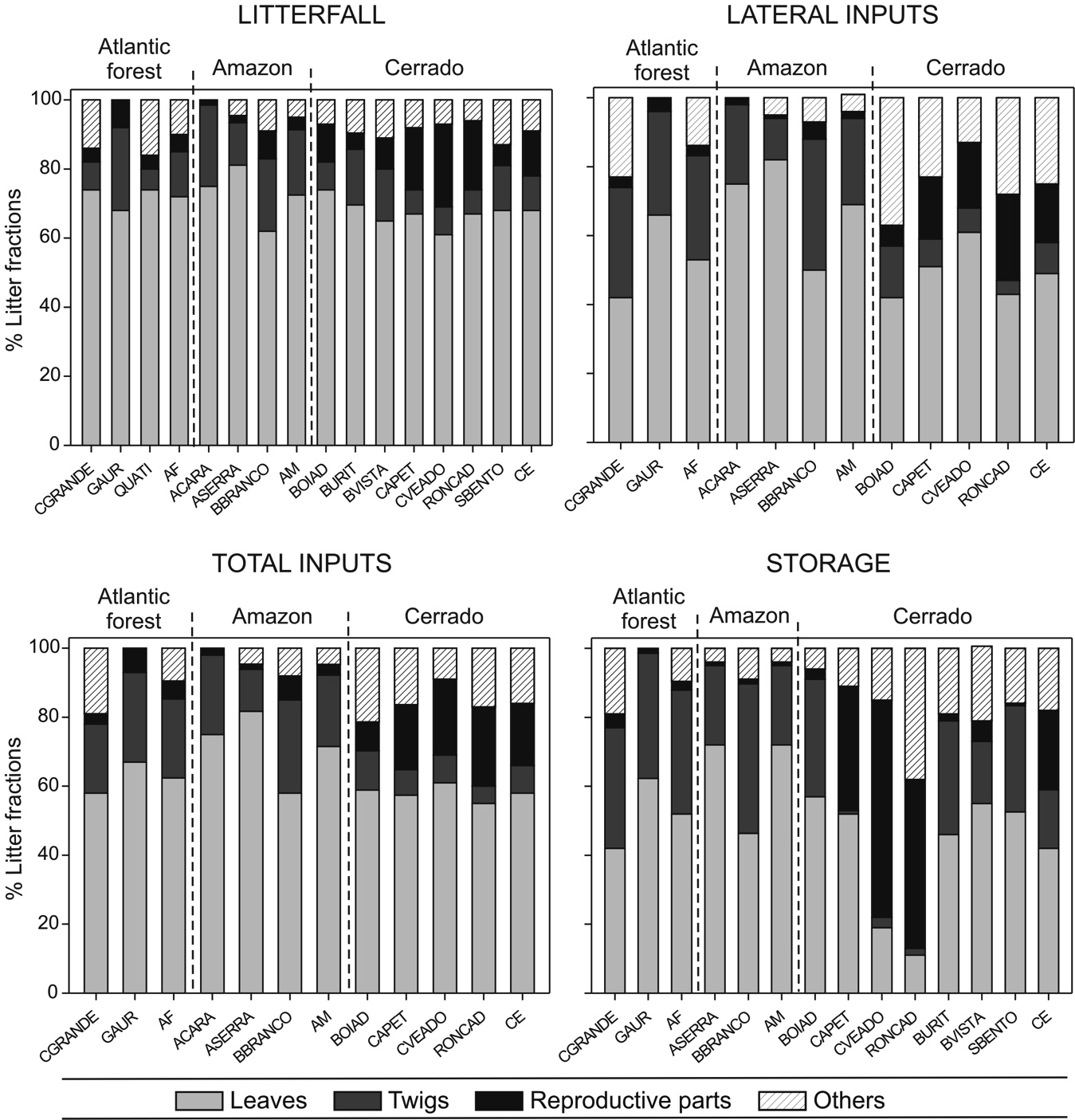


**Figure S3.** Proportion (%) of leaves, twigs, reproductive parts (flowers, fruits and seeds) and other unidentifiable litter parts of litterfall, lateral and total inputs (sum of litterfall and lateral inputs) to the stream, and storage in Atlantic forest, Cerrado and Amazon biomes.

Supplementary references

1 Lisboa, L. K., Lemes da Silva, A. L., Siegloch, A. E., Gonçalves, J. F. J. & Petrucio, M. M. Temporal dynamics of allochthonous coarse particulate organic matter in a subtropical Atlantic rainforest Brazilian stream. *Marine and Freshwater Research* **66**, 674-680 (2015).

2 Capellesso, E. S. Effects of forest structure on litter production, soil chemical composition and litter-soil interactions. *Acta Botanica Brasilica* **30**, 329-335 (2016).

3 Bambi, P. *et al.* Temporal and spatial patterns in inputs and stock of organic matter in savannah streams of central brazil. *Ecosystems* **20**, 757-768 (2017).

4 Zuur, A., Ieno, E. N., Walker, N., Saveliev, A. A. & Smith, G. M. *Mixed effects models and extensions in ecology with R*. (Springer Science & Business Media, 2009).
